# Genetic Counselors’ Experience with and Opinions on the Management of Newborn Screening Incidental Carrier Findings

**DOI:** 10.1007/s10897-018-0258-0

**Published:** 2018-04-23

**Authors:** Kristen Leppert, Katharine Bisordi, Jessica Nieto, Kristin Maloney, Yue Guan, Shannan Dixon, Alena Egense

**Affiliations:** 1 University of Maryland School of Medicine, Baltimore, MD 21201 USA; 2 Johns Hopkins University, Baltimore, MD 21287 USA; 3 GeneDx, Gaithersburg, MD USA

**Keywords:** Newborn screening, Carrier status, Incidental findings, Genetic counselors, Heterozygous, Policy

## Abstract

Newborn screening (NBS) is a public health program whose aim is to identify infants who will be clinically affected with a serious metabolic, genetic, or endocrine disorder; however, the technology utilized by many NBS programs also detects infants who are heterozygous carriers for autosomal recessive conditions. Discussion surrounding disclosure of these incidental carrier findings remains controversial. The purpose of this study was to assess genetic counselors’ attitudes about disclosure of carrier status results generated by NBS and to gather data on their experiences with incidental carrier findings. An electronic survey was distributed to genetic counselors of all specialties via the NSGC listserv, and a total of 235 survey responses were analyzed. Quantitative data were analyzed using IBM SPSS v24, and qualitative data were manually analyzed for thematic analysis. Results show that the counselor participants were overall in favor of routine disclosure. Those with experience in NBS were much more likely to strongly agree with one or more reasons *for disclosure* (*p* < 0.001), whereas those with five or fewer years of experience were more likely to strongly agree with one or more reasons *for non-disclosure* (*p* = 0.031). Qualitative analysis identified key motivating factors for disclosure, including helping parents to understand a positive screen, parents may otherwise be unaware of reproductive risk and they may not otherwise have access to this information, and, while genetic testing is inherently a complex and ambiguous process, this does not justify non-disclosure. The main motivating factor for non-disclosure was the need for better counseling and informed consent. The data suggest that implementation of an “opt-in/out” policy for parents to decide whether or not to receive incidental findings would be beneficial. The results of this study support the continued disclosure of incidental carrier findings; however, additional research is necessary to further determine and implement the most effective disclosure practices.

## Introduction

Newborn screening is a state public health program which aims to screen all infants shortly after birth for a variety of serious genetic, metabolic, and endocrine disorders in order to identify affected infants early in life and begin treatment and management to reduce morbidity and mortality (“Baby’s First Test” 2015). However, many newborn screening protocols also reliably detect heterozygous carriers of autosomal recessive conditions, such as cystic fibrosis or sickle cell disease (Miller et al. 2009). In 1994, the Institute of Medicine published a report recommending that parents be informed in advance of the possibility of incidental carrier findings and that this information be revealed to parents at their request, in the context of genetic counseling (Andrews et al. 1994). The topic of disclosure remains controversial, but current practice is to provide carrier status information to parents following newborn screening (Miller et al. 2009; Ross 2010). Method of notification, who is notified, and how aggressively identification is pursued vary widely across programs (Lang and Ross 2010).

Genetic counselors are often involved in the follow-up of abnormal newborn screening results, such as when follow-up diagnostic testing is required to distinguish an affected individual from a false positive or from a carrier infant. Genetic counseling for parents may be necessary to explain the significance of carrier status for the child, themselves, siblings of the carrier infant, and other family members (Moseley et al. 2013). As part of the healthcare team for infants and families in these situations, management of incidental carrier findings from newborn screening is an issue which impacts the field of genetic counseling (Hayeems et al. 2008; Kavanagh et al. 2008. This is a relevant issue through both its implications for use of and access to genetic counseling services for these families, as well as for genetic counselors following best practice guidelines when these situations occur (Noke et al. 2014).

### Purpose of the Study

The purpose of this study was to evaluate genetic counselors’ experience with and opinions on how incidental carrier findings should be managed. Genetic counselors of all clinical specialties were surveyed in order to (1) assess genetic counselors’ attitudes about the disclosure of carrier status results generated by newborn screening, (2) gather data on genetic counselors’ past experiences with incidental carrier findings and perceived impact of disclosure and knowledge of carrier status on the family, and (3) determine genetic counselors’ views on the future of newborn screening methods which will reduce or increase the number of carrier infants identified.

## Methods

### Overview

With approval from the University of Maryland Baltimore Institutional Review Board, this mixed methods study was conducted with respondents recruited from the National Society of Genetics Counselors’ membership from September to November of 2016.

### Participants

The sample consisted of genetic counselors within all fields of practice. Genetic counselors were chosen as previous studies have looked only at other healthcare providers such as pediatricians or have studied genetic counselors only as part of a larger group of genetics professionals. Counselors were not excluded from this study based on specialty or clinical involvement, as all genetic counselors are familiar with the complexities and ethical dilemmas involved in genetic testing (including genetic testing in minors and informed consent) and can therefore provide valuable responses to survey questions concerning attitudes towards disclosure and future newborn screening methods.

### Instrumentation and Procedures

An investigator-derived survey was developed as part of a master’s thesis requirement and modified by a committee of genetic counselors with experience in newborn screening follow-up and research. The electronic survey was created using SurveyMonkey and distributed to members of the National Society of Genetic Counselors via an e-mail blast through the NSGC listserv on September 22, 2016. A reminder e-mail was sent to participants on October 24, 2016, and the survey was closed to responses on November 25, 2016. The majority of the questionnaire (31 items) was the same for all participants. This included demographic information, state-specific information on the participant’s newborn screening program, assessment of participant’s agreement/disagreement with seven statements in favor of disclosure and nine statements in favor of non-disclosure, reasons for supporting disclosure and non-disclosure, and past experience with disclosure. Those participants who reported experience with disclosing newborn screening incidental carrier findings to parents were directed to additional questions regarding their past experiences in greater detail. The structure of the survey was designed to collect a large amount of quantitative data from counselors across specialties, as well as enriched qualitative data from a smaller subset of the sample population with experience in newborn screening follow-up for analysis and comparison.

### Data Analysis

Data from participant questionnaires were downloaded from SurveyMonkey. Quantitative data were analyzed using IBM SPSS version 24. For statistical analysis, each question was assessed based on the number of participants who completed each individual question, not based on the total survey participant number. Likert scales were collapsed into three categories (agree, no opinion, and disagree). Descriptive statistics were computed for all variables measured, including frequency counts and percentages. A chi-square test was used to determine differences in categorical variables. A probability level of < 0.05 was used to determine statistical significance. Responses to open-ended questions were manually analyzed by the first author for thematic analysis. A major theme was defined as being reported by greater than or equal to ten respondents; a minor theme was defined as anything reported by five to nine respondents; and other notable points reported by less than five respondents are also included. Additionally, quotations from participants that highlighted certain recurring themes were included as illustrative examples.

## Results

### Respondent Characteristics

Assuming approximately 2900 members in NSGC (estimated membership as of 2014) and a 15% estimated response rate, the target response number was 435 completed surveys. A total of 236 responses were received. One response submitted by a genetic counseling student was removed, as this participant was not a practicing genetic counselor and therefore was not eligible for this study. Two hundred thirty-five responses were analyzed, and each question was assessed based on the number of participants who completed the individual question. Demographic data about the sample are displayed in Table 1. Approximately 96% (*n* = 224) of respondents were female, and 3.8% (*n* = 9) were male, similar to that reported in the NSGC Professional Status Survey (PSS) of 96% female and 4% male. Nearly half of respondents (48.9%, *n* = 114) were under the age of 30 years, and three quarters were under 40 years. Two thirds of counselors reported having between 0 and 6 years of experience (66.3%, *n* = 156). Both of these findings represent a greater predominance of younger genetic counselors than reported in the PSS. Genetic counselors with greater than 12 years of experience and those over age 35 years were under-represented in the present sample. Eighty-one percent (*n* = 191) of counselors participated directly in patient care as a regular part of their job, and the remaining 19% (*n* = 44) were non-clinical counselors who do not regularly provide patient care, as compared to 69% clinical and 23% non-clinical according to the PSS (with 8% of PSS respondents recording no answer). Counselors were asked where they practice, and results are reported as defined by NSGC regions. Seven out of ten states with the greatest number of genetic counselors corresponded to those found in the PSS. Additionally, participants were asked to define their primary work setting, which is reported for both clinical and non-clinical counselors. Overall, primary work settings were similar to those found in the PSS.

**Table 1:** Participant demographics

Characteristic	Number	Percent	Characteristic	Number	Percent
Years experience			Age		
0–1 year	72	30.6	20–24	21	9
2–6 years	84	35.7	25–29	93	39.9
7–11 years	36	15.3	30–34	57	24.5
12–16 years	20	8.5	35–39	26	11.2
17–21 years	10	4.3	40–44	12	5.2
22–26 years	6	2.6	45–49	7	3
27–31 years	5	2.1	50–54	8	3.4
32–36 years	2	0.9	55–59	4	1.7
Valid total	235	100	60–64	4	1.7
Missing	0		65–70	1	0.4
Total	235		Valid total	233	100
			Missing	2	
Sex			Total	235	
Female	224	95.7			
Male	9	3.8	Sees patients?		
Prefer not to answer	1	0.4	Clinical counselor	191	81.3
Valid total	234	100	Non-clinical counselor	44	18.7
Missing	1		Valid total	235	100
Total	235		Missing	0	
			Total	235	
Primary work setting (non-clinical)					
Commercial diagnostic laboratory	22	51.2	Primary work setting (clinical)		
Academic Medical Center	13	30.2	Academic Medical Center	81	42.6
Private hospital/medical facility	2	4.7	Public hospital/medical facility	47	24.7
Public hospital/medical facility	0	0	Private hospital/medical facility	40	21.1
State health department	0	0	State health department	10	5.3
Other non-clinical counseling setting:	6	14	Commercial diagnostic laboratory	6	3.2
Research	2		Other clinical counseling setting:	6	3.2
Non-profit	2		Non-profit	4	
Gamete facility	1		NBS program	1	
Health plan	1		Department of Veterans Affairs	1	
Valid total	43	100	Valid total	190	100
Missing	192		Missing	45	
Total	235		Total	235	
NSGC regions			Narrow specialty categories		
Region 1	8	6.1	Prenatal	53	22.6
Region 2	23	17.6	Pediatric	56	23.8
Region 3	17	13	Cancer	40	17
Region 4	42	32.1	Other	86	36.6
Region 5	20	15.3	Valid total	235	100
Region 6	18	13.7	Missing	0	
Other			Total	235	
London, UK	3	2.3			
Australia					
Germany					
Valid total	131		Experience with either NBS follow-up or disclosure of incidental carrier findings		
Missing	104		No	107	45.5
Total	235		Yes	128	54.5
			Valid total	235	100
			Missing	0	
			Total	235	

Choices for specialty categories were defined from those published in the NSGC PSS. Respondents reported both primary and secondary specialty areas. Specialty categories were then grouped into four broad categories based on counselor responses: prenatal (including infertility, ART/IVF, PGD, prenatal multiple marker screening, and teratogens), pediatric (including newborn screening follow-up and metabolic disease), cancer, and other (encompassing all other specialty categories reported). The number of respondents in each category is reported in Table 1 under the heading “broad specialty.” Responses about current newborn screening practices as reported by counselors are displayed in Table 2. Approximately 80% (*n* = 184) of counselors reported that their state’s newborn screening program does identify carriers of autosomal recessive conditions. Over half (60.8%, *n* = 110) were unsure if their state currently has a protocol in place for follow-up after an infant is identified as a carrier. Of those who were aware of whether or not a protocol was in place, 5% (*n* = 9) said no, and the remaining 34.3% (*n* = 62) said yes. Counselors were also asked if parents are notified of incidental carrier results (discovered by the initial screen itself or by follow-up diagnostic testing) for all, some, or none of the conditions on the newborn screen. Of the 181 respondents who answered this question, 58% (*n* = 105) were unsure, and 1.1% (*n* = 2) said no. Of those who answered that their state does disclose carrier findings, 30.4% (*n* = 55) said carrier status is disclosed for *all* conditions, and 10.5% (*n* = 19) said carrier status is disclosed to parents for some but not all conditions.

**Table 2:** Current NBS practices reported by participants

	Number	Percent
Does your state’s newborn screening program identify carriers of one or more genetic conditions via the initial screen and through follow-up diagnostic testing for an abnormal screen (for example: sickle-cell, cystic fibrosis, galactosemia, VLCAD deficiency)?		
Yes	184	79.7
No	3	1.3
Unsure	44	19
Total	231	100
Missing	4	
Total	235	
In your state, are parents notified of carrier status information discovered by follow-up diagnostic testing after an abnormal screen result?		
Yes—all	55	30.4
Yes—some	19	10.5
No	2	1.1
Unsure	105	58
Total	181	100
Missing	54	23
Total	235	100
Does your state’s newborn screening program have a protocol in place?		
Yes	62	34.3
No	9	5
Unsure	110	60.8
Total	181	100
Missing	54	
Total	235	

### Attitudes About Disclosure: Quantitative

**Fig. 1:**
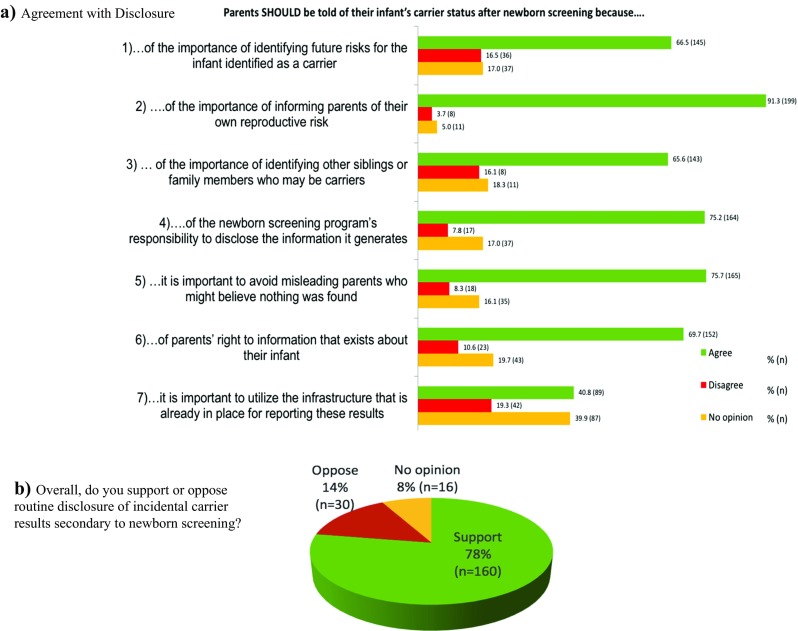
**a** Agreement with disclosure. **b** Overall, do you support or oppose routine disclosure of incidental carrier results secondary to newborn screening?

**Fig. 2:**
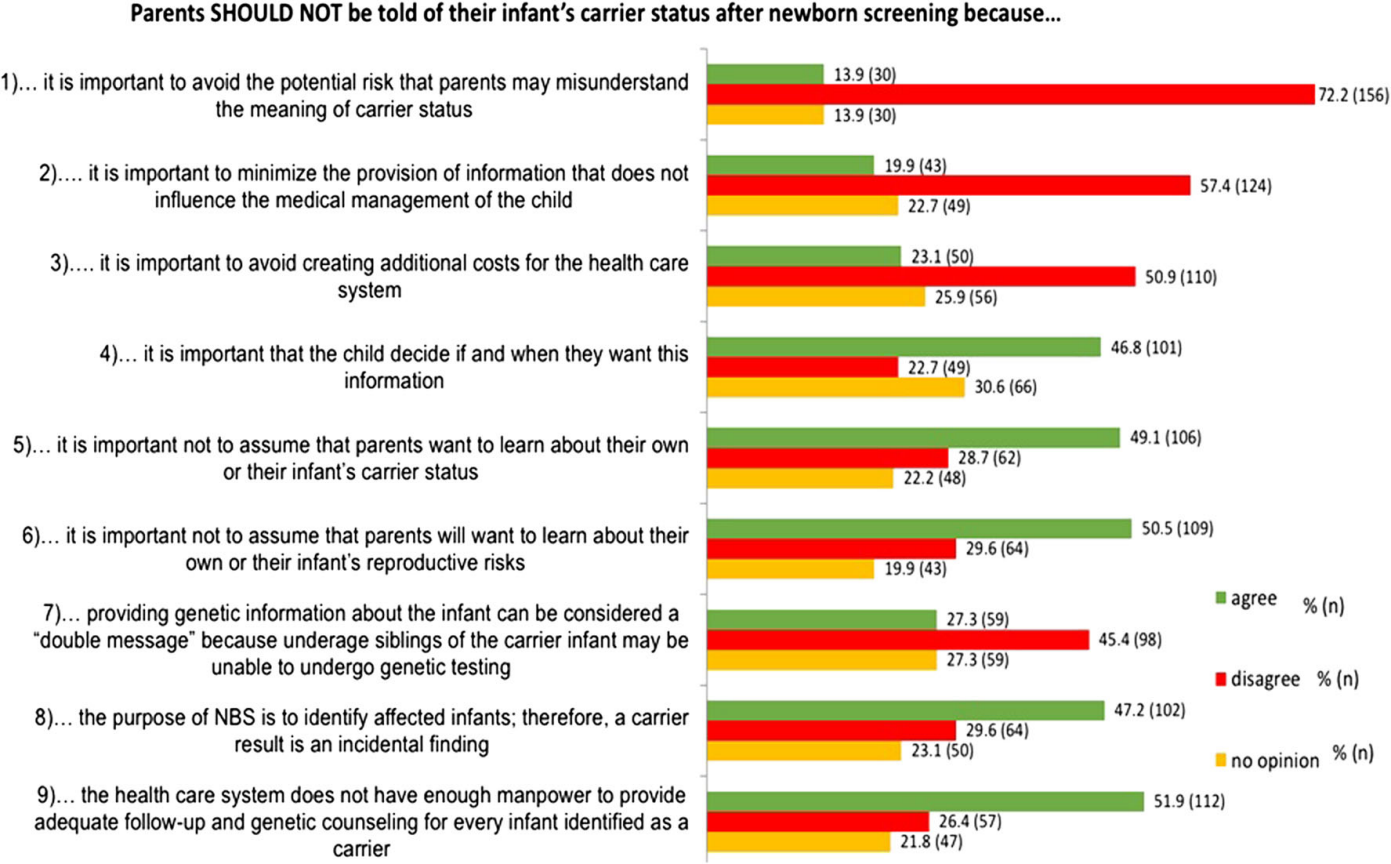
Agreement with non-disclosure

Overall support for disclosure was assessed based on participant agreement with seven statements of motivation for favoring disclosure using a five-point Likert scale. Overall support for non-disclosure was similarly assessed based on participant agreement with nine reasons favoring non-disclosure. Genetic counselors’ agreements with these statements are reported in Figs. 1a and 2, respectively.

The most highly agreed with reasons for disclosure were the importance of informing parents of their own reproductive risk (91.3% agreement, *n* = 199), the importance of avoiding misleading parents who might believe nothing was found (75.7% agreement, *n* = 165), and the newborn screening program’s responsibility to disclose the information it generates (75.2% agreement, *n* = 164). Disagreement did not outweigh agreement for any of the seven reasons. The most highly disagreed with reason for disclosure was the importance of utilizing the infrastructure that is already in place for reporting these results (19.3%, *n* = 42). However, nearly 40% (*n* = 87) did not have an opinion on this reason. Of all reasons for disclosure, this reason had the greatest variability in counselor responses.

The most highly agreed with reasons for non-disclosure (where agreement did outweigh disagreement) were the statements that the healthcare system does not have enough manpower to provide adequate follow-up and genetic counseling for every infant identified as a carrier (51.9%, *n* = 112); it is important not to assume that parents will want to learn about their own or their infant’s reproductive risks (46.4%, *n* = 109); it is important not to assume that parents want to learn about their own or their infant’s carrier status (45.1%, *n* = 106); the purpose of NBS is to identify affected infants and, therefore, a carrier result is an incidental finding (47.2%, *n* = 102); and it is important that the child decide if and when they want this information (46.8%, *n* = 101). The most highly disagreed with reasons for non-disclosure (where disagreement did outweigh agreement) included that it is important to avoid the potential risk that parents may misunderstand the meaning of carrier status (72.2%, *n* = 156); it is important to minimize the provision of information that does not influence the medical management of the child (57.4%, *n* = 124); it is important to avoid creating additional costs for the healthcare system (50.9%, *n* = 110); and providing genetic information about the infant can be considered a “double message” because underage siblings of the carrier infant may be unable to undergo genetic testing (45.4%, *n* = 98).

**Fig. 3:**
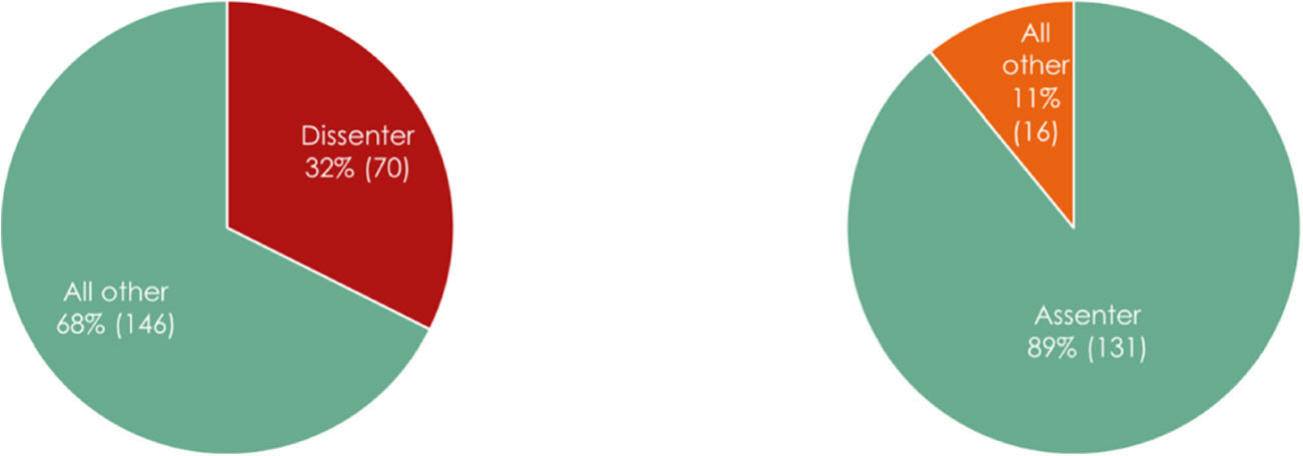
Left: Dissenting genetic counselors (strongly agreed with one or more reasons for non-disclosure) - percent (*n*); Right: assenting genetic counselors (strongly agreed with one or more reasons for disclosure) - percent (*n*)

An individual was defined as a dissenter if they strongly agreed with one or more reasons for non-disclosure. An individual was defined as an assenter if they strongly agreed with one or more reasons for disclosure. The vast majority (89%, *n* = 131) of respondents fell into the assenter category. Thirty-two percent (*n* = 70) were defined as a dissenter. These measures are reported in Fig. 3. Of note, it is possible to be defined as both an assenter and a dissenter, as these are independent measures based on responses to separate questions. Therefore, this finding should be interpreted as only one piece of data and not representative of those who do or do not support disclosure. For this reason, counselors were also asked if, overall, they support or oppose routine disclosure. These responses (depicted in Fig. 1b) show that 78% (*n* = 160) of counselors supported disclosure, 14% (*n* = 30) opposed disclosure, and 8% (*n* = 16) had no opinion.

Respondents who reported experience with either newborn screening or disclosure of incidental carrier findings were overall much more likely to be defined as an assenter (*p* < 0.001).

Specifically, those with experience were more likely to *agree* with reason #6 for disclosure (because of parents’ right to information that exists about their infant, *p* = 0.007). They were also more likely to *disagree* with reasons #3 (it is important to avoid creating additional costs for the healthcare system, *p* = 0.048), #4 (it is important that the child decide if and when they want this information, *p* = 0.047), #5 (it is important not to assume that parents want to learn about their own or their infant’s carrier status, *p* = 0.029), and #9 (the healthcare system does not have enough manpower to provide adequate follow-up and genetic counseling for every infant identified as a carrier, *p* = 0.021) for non-disclosure.

Overall support of disclosure between broad specialties produced a significant *p* value when compared utilizing a chi-square test (*p* = 0.042). However, when compared using the Kruskal-Wallis test for independent samples, the difference was not statistically significant (*p* = 0.538). Therefore, overall support of disclosure did not differ based on broad specialty experience between any of the two groups based on a non-normal distribution.

Those with five or fewer years of experience were more likely to be dissenters (*p* = 0.031). Those with five or fewer years of experience were more likely to agree with reason #4 (it is important that the child decide if and when they want this information, *p* = 0.001), #5 (it is important not to assume that parents want to learn about their own or their infant’s carrier status, *p* = 0.007), and #6 (it is important not to assume that parents will want to learn about their own or their infant’s reproductive risks, *p* = 0.007) for non-disclosure. Overall, those with five or fewer years of experience were more likely agree with reasons for non-disclosure (*p* = 0.009).

### Attitudes About Disclosure: Qualitative

In addition to quantitative assessment of agreement with disclosure versus non-disclosure, the survey also included several open-ended questions which allowed counselors to elaborate further on their stance and to contextualize their views within their experiences and practice (Table 3). These results are not intended to be representative of the views of all genetic counselors but rather to provide an opportunity for counselors to express a more detailed perspective, especially as this is a complicated issue and a final opinion may be based on many contributing factors. Answers to open-ended questions were not required in order to move on with the survey. Free text responses were manually analyzed for thematic analysis by one author and assessed for commonality.

**Table 3:** Quantitative data thematic analysis

Overall, do you support or oppose routine disclosure of incidental carrier results secondary to newborn screening, and why? General themes Major: ▪ Incidental carrier findings should be optional and only withheld from parents who choose to “opt-out” Minor: ▪ Many respondents support disclosure with reservations or under circumstances/conditions	
Motivation for disclosure Major: ▪ Help parents understand a positive screen ▪ Help identify affected infants ▪ May impact health of carrier infant Minor ▪ Save money and avoid re-screening ▪ May get new information	Motivation for non-disclosure Major: ▪ Need better counseling/education/informed consent
Other notable points: ▪ Increase awareness of NBS ▪ Creates mistrust ▪ Legal obligation	

First, counselors were asked to elaborate upon why they either support or oppose routine disclosure. One hundred fifty-one open-ended responses were received, and themes from these responses are summarized in Table 3. A major theme was defined as being reported by greater than or equal to ten respondents; a minor theme was defined as anything reported by five to nine respondents. Other notable points reported by less than five respondents are also listed. The most frequently mentioned theme is that counselors felt that incidental carrier status results should always be reported to parents, unless they choose to opt out, similar to the practice of newborn screening itself.

Some key motivating factors for disclosure include the following: (1) Helping parents to understand a positive screen: *It is not truly incidental if it explains an abnormal NBS (reduced enzyme activity in carriers).*—participant #83; (2) parents may otherwise be unaware of reproductive risk, and for some couples, this may be the only avenue through which they have access to this information if prenatal or preconception carrier screening was not available: *Historically medically underserved patients are those least likely to have access to their own carrier testing, so receiving this sort of information through a public health venture like NBS may help alleviate some disparities in access to carrier testing.*—participant #142; and (3) genetic testing and counseling is inherently a complex and sometimes ambiguous process; however, this does not justify non-disclosure: *Statistically a carrier-carrier couple is more likely to have a carrier child than an affected child. …Avoiding doing something because the effort is “hard” or “costly” risks weakening the support for doing it all. Contact to carrier families may be of lower priority but not doing it at all risks undermining the support behind NBS as a whole.*—participant #182. The main motivating factor for non-disclosure was the need for better counseling and obtaining informed consent from parents: *The purpose of a wide screening program without proactive informed consent is to prevent deadly diseases of infancy. Carrier screening is genetic testing without informed consent of the family or patient.—*participant #219.

Lastly, counselors also shared other important insights into the ethical dilemma of what to do with incidental carrier findings, including that (1) there is often misunderstanding between screening and diagnosis; (2) ultimately, it all comes down to the quality of counseling that parents receive; (3) discordant results cause additional confusion; and (4) genetic counselors are in high demand, and counseling parents about carrier status information that is likely not of clinical utility may not be the most efficient use of limited resources: *Mostly because this is a screening test, and follow-up testing will need to be performed before or after obtaining a “positive” result, requiring more cost, time, and genetics expertise and infrastructure that could be used, at this time, more effectively in other genetics clinics.*—participant #85.

### Opinions on the Future of Newborn Screening Methods

All participants received questions concerning their stance regarding the future of newborn screening. Table 4 reports the results of overall agreement with various screening methodologies. Overall, 41.7% (*n* = 86) of participants would support the implementation of molecular testing methods in newborn screening programs, even if this meant identification of more carriers and the state required that carrier status must be disclosed. Thirty-two percent (*n* = 66) were opposed to these molecular methods, and the remainder (26.2%, *n* = 54) answered “no opinion.” One hundred fifteen respondents provided open-ended responses to expand upon their opinion. When asked if they would support testing methods which avoid carrier detection, 49% (*n* = 101) supported, 15% (*n* = 31) opposed, and 35.9% (*n* = 74) answered no opinion. One hundred one respondents provided an open-ended response to this question. Of note, for both questions, several participants stated that they would need to know more about the sensitivity, specificity, and cost of the testing. Additionally, participants also voiced concerns about the feasibility of using methods that avoid carrier detection completely, as this would require increasing the threshold or screening cutoff, which would consequently result in missing affected infants in order to avoid detecting heterozygous carriers. One of the most frequently cited reasons in support of molecular testing is that it may provide more accurate results in a timely fashion and ultimately improve patient care.

**Table 4:** Participant opinions on newborn screening methodology

	Number	Percent
Overall, would you support or oppose the implementation of molecular testing methods into all newborn screening programs, if it would result in more carriers being identified and if states would require disclosure of these results?		
Support	86	41.7
Oppose	66	32
No opinion	54	26.2
Valid total	206	100
Missing	29	
Total	235	
Would you support or oppose the implementation of testing methods which do not detect carrier infants into newborn screening programs?		
Support	101	49
Oppose	31	15
No opinion	74	35.9
Valid total	206	100
Missing	29	
Total	235	

Conversely, there were many arguments made against the implementation of molecular testing methods, including the following: (1) the issue of variants of uncertain significance was a strong motivating factor; (2) low cost/benefit ratio and inefficient use of resources; (3) current infrastructure cannot support this practice; (4) may result in an overall decrease in uptake of newborn screening; and (5) there is no sufficient research/data available to support this practice at this time. Overall, participants felt strongly that the best test for each disorder deemed appropriate for testing should be employed.

### Counselor Experiences with Disclosure

Counselors were asked to report whether or not they had seen evidence of harms from non-disclosure versus disclosure actually occurring in practice. Of the 206 counselors who answered about harms resulting *from disclosure*, approximately one third reported that they are not a clinical counselor (32.5%, *n* = 67) and therefore could not comment. Nearly 40% (*n* = 82) answered no; however, 27.7% (*n* = 57) responded yes; they had witnessed harms resulting from disclosure and provided an open-ended response to elaborate on their experience. The most commonly reported harms were unjustified parental anxiety and misunderstanding. This misunderstanding can include comprehension of the meaning of carrier status, the implications for the health of the child, and implications for the reproductive health of parents and other family members. Additionally, counselors reported concern for autonomy of the infant and that identification of carriers is not the intended purpose of this public screening program. Four participants cited specific cases where disclosure of carrier findings led to the discovery of non-paternity.

Along with the previously reported harms mentioned above, counselors also gave examples of harms they have observed in practice that were not known to be previously reported in the literature, including the following: (1) initiating unnecessary treatment/intervention based on misunderstood information (this could be on the part of the parents or the provider themselves; for example, modifying an infant’s diet based on a carrier result for a metabolic disorder) and (2) carrier results leading to molecular testing, which frequently detects variants of uncertain significance and leads to an increase parental anxiety in situations where it is unclear if the child is a carrier or truly affected.

Conversely, when asked if they had seen harms resulting *from withholding carrier status*, 31.6% (*n* = 65) chose no comment, and 50% (*n* = 103) reported that they had not experienced harms. The remaining 18.4% (*n* = 38) reported that they have observed harms from non-disclosure and provided open-ended responses detailing these experiences. Based on respondent answers, many of the harms found in the literature and cited earlier in this publication do indeed occur in practice. In particular, several counselors cited cases where parents were unaware of their reproductive risk, resulting in the birth of a future, affected child. Counselors also provided specific, novel examples of harms of non-disclosure and benefits of disclosure, including the following: (1) parental mistrust after the first child’s carrier status was not disclosed, but a subsequent child’s carrier status was disclosed; (2) parents may not have access to carrier screening otherwise; and (3) identifying an older, mildly affected sibling.

**Fig. 4:**
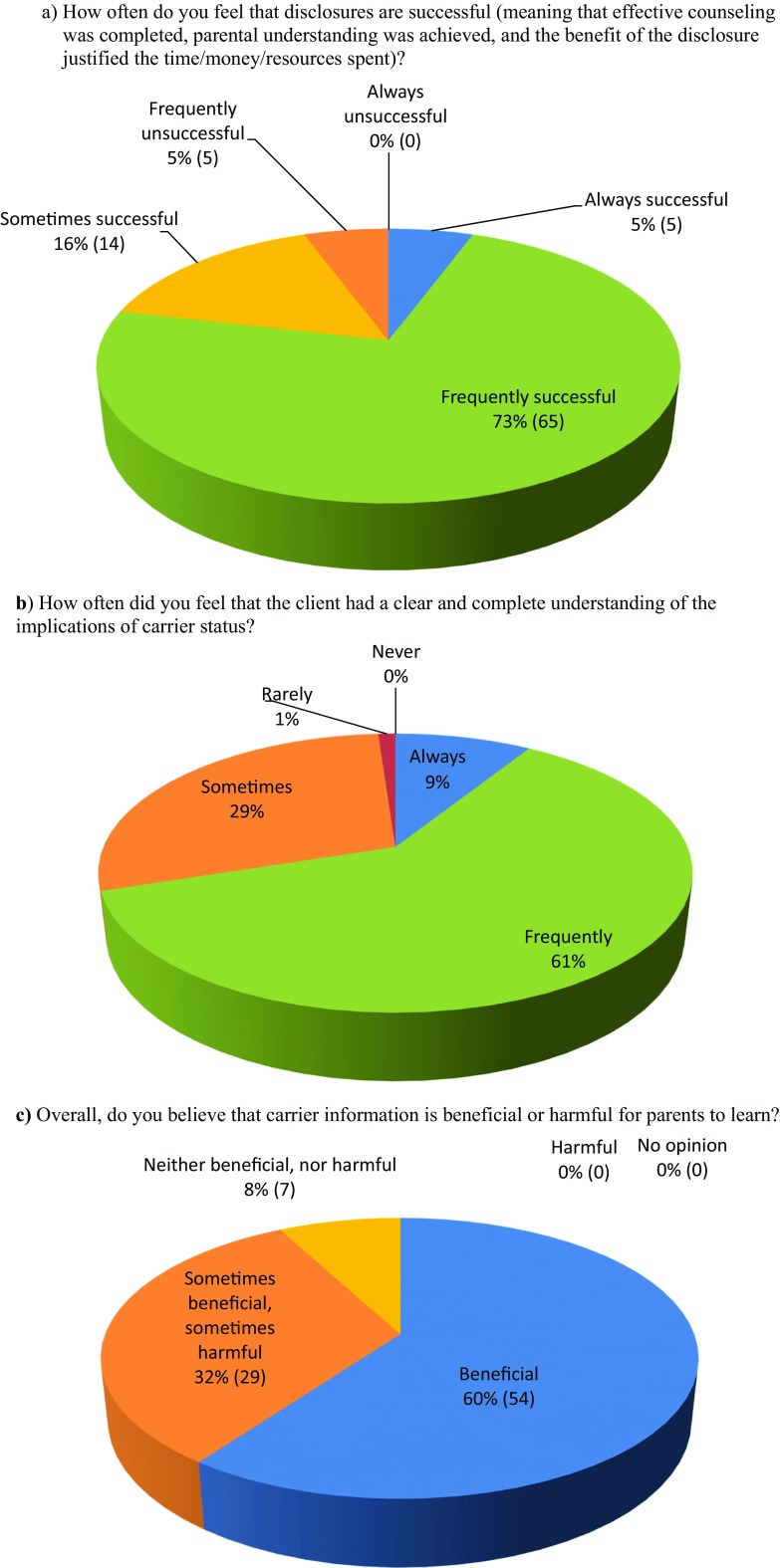
Perceived effectiveness of carrier status disclosure. **a** How often do you feel that disclosures are successful (meaning that effective counseling was completed, parental understanding was achieved, and the benefit of the disclosure justified the time/money/resources spent)? **b** How often did you feel that the client had a clear and complete understanding of the implications of carrier status? **c** Overall, do you believe that carrier information is beneficial or harmful for parents to learn?

Counselors who reported previous experience with disclosure of newborn screening incidental carrier findings (*n* = 90) were directed to an additional subset of questions in order to assess the perceived impact and effectiveness of disclosure counseling with parents of carrier infants. Participants were asked to rank how frequently parents expressed a variety of both positive and negative emotions during or shortly after disclosure of incidental findings. Based on these results, positive emotions overall outweighed negative emotions (data not shown). The most commonly reported negative emotions were anxiety and confusion. The most commonly reported positive reaction was comprehension, followed by relief and reassurance. Participants were also asked to rate how often they felt clients had a clear and complete understanding of the implications of carrier status, how often they felt disclosures were successful, and whether they felt this information was overall beneficial or harmful for parents to learn. These responses are reported in Fig. 4a–c. Overall, reported understanding and success were very high, with 70% of participants reporting that understanding occurred frequently to always, and 78.6% reporting success occurred frequently to always. The majority of participants (60%, *n* = 54) felt that carrier status is beneficial for parents to learn, no participants responded that it is overall harmful for parents to learn, and 32.3% (*n* = 29) responded “sometimes beneficial, sometimes harmful.”

## Discussion

### Practice Implications

A previous study by Miller et al. (2009) found that healthcare providers overall favored disclosure of newborn screening incidental carrier findings; however, genetics professionals (including genetic counselors) were one half to seven times more likely to disagree or strongly disagree with reasons for disclosure. Miller and colleagues recommended that this minority dissenting group be studied in greater depth as part of a thorough evaluation to determine how newborn screening incidental carrier findings should be managed. The current study attempted to address this previous knowledge gap and found that not only did the majority of genetic counselor participants favor disclosure, but also those with newborn screening follow-up and/or experience with disclosure of incidental carrier findings were actually more likely to agree with disclosure. These findings suggest that, while genetic counselors are able to well articulate critical concerns which need to be addressed in the disclosure of carrier findings, overall sentiment favors the disclosure of these findings.

The genetic counselors’ opinions reflect the delicate balance between the duty to inform parents of their own and their child’s reproductive risk, while also not assuming that parents will always want to learn this information. The results of this study suggest that the implementation of an “opt-in/opt-out” policy for parents to make an informed decision about whether or not to receive incidental findings may be beneficial. However, this option may not be feasible due to limitations including a shortage of genetic counselors, who are ideal healthcare professionals to provide counseling and education, and obtain informed consent/dissent from parents.

While implementation of this particular solution may not be feasible at the present time, the authors of the present study suggest some alternatives. First, parents could be provided with an informational brochure or access to online resources to help inform and educate them on the newborn screening process and potential results, including positive, negative, and inconclusive findings during the prenatal period. These educational materials should include discussion about the basics of autosomal recessive inheritance and the possibility of incidental carrier findings. Second, as the responsibility of counseling about incidental carrier findings often falls to pediatricians or other healthcare providers (Farrell et al. 2001; Farrell & Christopher 2013; Stark et al. 2011), it may be beneficial for genetic counselors to help provide education to these providers on how to effectively counsel regarding newborn screening findings and how to minimize harms resulting from disclosure. This could be accomplished through a variety of formats, including educational sessions, online webinars, providing counselor-developed teaching aids to physicians, and establishing a peer mentorship program so that counselors can be available to serve as a resource or sounding board for other providers in cases where there are unusual circumstances or a question about how to counsel a family.

Until such time that alternative approaches such as those mentioned above may be developed and implemented, the results of this study suggest that genetic counselors overall support disclosure of incidental carrier findings. The present results further suggest that while harms may result from disclosure, particularly when a genetic counselor is not involved, the potential harms, such as violation of infant autonomy, unjustified parental anxiety, and misunderstanding of carrier status, do not seem to outweigh the benefits of disclosure.

### Study Limitations

This study has some inherent limitations which should be taken into account when interpreting the findings. The sample population was accessed through the National Society of Genetic Counselors (NSGC) e-mail listserv; therefore, counselors who were not NSGC members at the time of the survey were not included. The sample size for this study fell below the target response rate and does not necessarily represent all genetic counselors. The sample population also may have been affected by ascertainment bias, as counselors with experience in newborn screening follow-up or those with particularly strong opinions regarding disclosure may have been more likely to complete the survey. Another limitation is that not all participants answered every question. For several questions concerning newborn screening practices, a large percentage of counselors selected “unsure” or no opinion, which may have limited statistical analysis and power, or may have skewed the data. Genetic counselors were asked about protocols pertaining to reporting of newborn screening incidental findings in the state where they practice. These responses are solely based on genetic counselor report, and were not able to be cross-checked with the protocol of each state. Therefore, these results should be interpreted as genetic counselors’ perceptions of the result disclosure process, and not necessarily the actual protocol in any given state. This lack of context for these results is another limitation of this study. Not every relevant topic concerning newborn screening findings was included in the questionnaire. As with any survey-based study, questions may have been misinterpreted or interpreted differently among participants due to the wording. Finally, a number of univariate tests were conducted without controlling for familywise error rate. Although acceptable in an exploratory study, it is possible that some of the statistically significant findings are due to chance.

### Research Recommendations/Future Directions

This study’s results are congruent with previous published reports that suggest that similar harmful effects are experienced as a result of routine disclosure of newborn screening incidental carrier findings. For this reason, future research should explore how to minimize these harms. Such research would inform education for providers on how to counsel parents about the meaning of possible carrier findings in an appropriate and accurate way at institutions where genetic counselors are not available to meet with every family. Additional long-term follow-up studies of parents’ views on disclosure of newborn screening incidental carrier findings are needed to further explore the parental perspective of benefit or harm of disclosure.

This study also found that counselors with five or fewer years of experience were more likely to agree with reasons for non-disclosure. This may be a result of differences in training as genetic counseling program curricula have evolved or increased comfort with ambiguity and patient anxiety as the world of genetic testing and knowledge continues to increase. Additional research is needed to determine why these experience differences exist, to further explore the motivations behind less experienced counselors’ opinions, and to help determine whether or not opinions regarding this issue may shift more towards favoring non-disclosure in the future.

Additionally, this topic should be revisited in future years, as counselor opinions may change or differ as more and newer conditions are added to newborn screening panels. While counselors may favor disclosure at this time, it is possible that if or when other conditions without effective intervention or treatment are screened for in the perinatal period, a reassessment of balancing the harms versus benefits of disclosure will be needed.

Lastly, with regard to the future of newborn screening methodology, this study found that 41.7% of counselors were in favor of implementing molecular testing methods into newborn screening. While this particular study was not focused on this question and did not clarify what conditions would be tested for using molecular testing, previous studies have considered genetic counselors’ views on this issue. One study by Nardini et al. (2014) found that 78.1% of counselors felt prepared to provide counseling for single gene sequencing for those conditions already included on newborn screening panels. However, only 21.5 and 17.9% felt prepared to counsel regarding whole exome sequencing and whole genome sequencing, respectively, as part of newborn screening results. While responses concerning the shift towards molecular-based newborn screening were divided, the trend seems to be that genetic counselors are becoming increasingly in favor of molecular testing methodologies being further incorporated into newborn screening. Therefore, more research will be needed to determine consumer perspectives, healthcare provider opinions, and how to implement effective pre- and post-test counseling, particularly at institutions where genetic counseling cannot feasibly be provided to every couple.

## Conclusions

Overall, genetic counselor agreement with routine disclosure of newborn screening incidental carrier findings was consistently high across different questions. This is consistent with findings of previous studies of healthcare providers and helps to clarify the viewpoint of genetics professionals on the dilemma of disclosure of newborn screening incidental carrier findings. The present findings suggest genetic counselors’ overall support disclosure of newborn screening incidental carrier findings. Those counselors with previous experience with newborn screening follow-up or disclosure of incidental carrier findings were more likely to support disclosure than other counselors. Counselors with fewer than 5 years of experience, however, were more likely to agree with reasons for non-disclosure. Anxiety and confusion were frequently reported emotions experienced by parents; yet, the majority of respondents disagreed that the risk for these emotions is a motivation for supporting non-disclosure. Genetic counselors are well equipped to educate parents about incidental carrier findings and reduce harms, but the shortage of genetic counselors supports the need for education of other providers on how to interpret and explain carrier findings in order to reduce potential harms.

## References

[CR1] Andrews LB, Fullarton JE, Holtzman NA, Motulsky G for the Institute of Medicine, Committee on Assessing Genetic Risks (1994). Assessing genetic risk: implications for health and social policy.

[CR2] Baby’s First Test (2015). Retrieved from http://www.babysfirsttest.org.

[CR3] Farrell MH, Christopher SA (2013). Frequency of high-quality communication behaviors used by primary care providers of heterozygous infants after newborn screening. Patient Education and Counseling.

[CR4] Farrell M, Certain L, Farrell P (2001). Genetic counseling and risk communication services of newborn screening programs. Archives of Pediatrics & Adolescent Medicine.

[CR5] Hayeems RZ, Bytautas JP, Miller FA (2008). A systematic review of the effects of disclosing carrier results generated through newborn screening. Journal of Genetic Counseling.

[CR6] Kavanagh PL, Wang CJ, Therrell BL, Sprinz PG, Bauchner H (2008). Communication of positive newborn screening results for sickle cell disease and sickle cell trait: variation across states. American Journal of Medical Genetics. Part C, Seminars in Medical Genetics.

[CR7] Lang CW, Ross LF (2010). Maternal attitudes about sickle cell trait identification in themselves and their infants. Journal of the National Medical Association.

[CR8] Miller FA, Hayeems RZ, Bombard Y, Little J, Carroll JC, Wilson B (2009). Clinical obligations and public health programmes: healthcare provider reasoning about managing the incidental results of newborn screening. Journal of Medical Ethics.

[CR9] Moseley K, Nasr S, Schuette J, Campbell A (2013). Who counsels parents of newborns who are carriers of sickle cell anemia or cystic fibrosis?. Journal of Genetic Counseling.

[CR10] Nardini MD, Matthews AL, Mccandless SE, Baumanis L, Goldenberg AJ (2014). Genomic counseling in the newborn period: experiences and views of genetic counselors. Journal of Genetic Counseling.

[CR11] Noke M, Wearden A, Peters S, Ulph F (2014). Disparities in current and future childhood and newborn carrier identification. Journal of Genetic Counseling.

[CR12] Oliver, S., Dezateux, C., Kavanagh, J., Lempert, T., & Stewart, R. (2004). Disclosing to parents newborn carrier status identified by routine blood spot screening. *The Cochrane Database of Systematic Reviews*, (4):CD003859. 10.1002/14651858.CD003859.pub2 PMC11491188 15495068

[CR13] Ross L (2010). Carrier detection in childhood: a need for policy reform. Genome Medicine.

[CR14] Stark AP, Lang CW, Ross LF (2011). A pilot study to evaluate knowledge and attitudes of Illinois pediatricians toward newborn screening for sickle cell disease and cystic fibrosis. American Journal of Perinatology.

